# Genome Sequence of Creatinine-Fermenting *Tissierella creatinophila* Strain KRE 4^T^ (DSM 6911)

**DOI:** 10.1128/genomeA.00051-17

**Published:** 2017-03-23

**Authors:** Heiko Nacke, Rolf Daniel, Anja Poehlein

**Affiliations:** Genomic and Applied Microbiology and Göttingen Genomics Laboratory, Institute of Microbiology and Genetics, Georg-August University, Göttingen, Germany

## Abstract

*Tissierella creatinophila* strain KRE 4^T^ (DSM 6911) is a strictly anaerobic, creatinine-fermenting, and creatine-fermenting organism, which has been isolated from sewage sludge. The draft genome consists of one circular chromosome (2.5 Mb) and harbors 2,533 predicted protein-encoding genes.

## GENOME ANNOUNCEMENT

The strictly anaerobic, Gram-positive, and non-spore-forming *Tissierella creatinophila* strain KRE 4^T^ is able to grow on creatinine or on creatinine plus formate as a sole source of carbon, nitrogen, and energy (1). Strain KRE 4^T^ was shown to degrade creatinine via creatine, sarcosine, and glycine to acetate, monomethylamine, ammonia, and carbon dioxide (1, 2). The growth of this strain with creatinine or creatine as substrates was selenium-dependent and stimulated by formate (1). *T. creatinophila* strain KRE 4^T^ (DSM 6911^T^) was isolated from sewage sludge and obtained from the German Collection of Microorganisms and Cell Cultures (DSMZ).

Chromosomal DNA of *T. creatinophila* strain KRE 4^T^ was isolated using the MasterPure complete DNA purification kit (Epicentre, Madison, WI, USA). The extracted DNA was used to generate Illumina shotgun paired-end sequencing libraries, which were sequenced with a MiSeq instrument and the MiSeq reagent kit version 3 as recommended by the manufacturer (Illumina, San Diego, CA, USA). Quality filtering using Trimmomatic version 0.32 (3) resulted in 2,751,284 paired-end reads. The assembly was performed with the SPAdes genome assembler software version 3.9.0 (4). The assembly resulted in 66 contigs (>500 bp) and an average coverage of 145-fold. The assembly was validated and the read coverage determined with QualiMap version 2.1 (5). The draft genome of *T. creatinophila* strain KRE 4^T^ (DSM 6911^T^) consists of a single chromosome (2,503,461 bp) with an overall G+C content of 30.68%. Automatic gene prediction and identification of rRNA and tRNA genes was performed using the software tool Prokka (6). The draft genome contained six rRNA genes, 49 tRNA genes, 1,839 protein-encoding genes with function prediction, and 694 genes coding for hypothetical proteins.

The conversion of creatinine to creatine is catalyzed by amidohydrolases. Analysis of the genome revealed that *T. creatinophila* strain KRE 4^T^ harbors two putative creatinine amidohydrolase genes. Furthermore, genes encoding cytosine deaminase, *N*-carbamoylsarcosine amidase, glycine reductase, as well as sarcosine reductase complex components A, B, and C, 5,10-methylenetetrahydrofolate reductase, methenyltetrahydrofolate cyclohydrolase, formate-tetrahydrofolate ligase, and formate dehydrogenase, which are associated with degradation of creatinine (1, 7), were identified. We also detected a gene encoding a D-hydantoinase-like protein forming a cluster with the gene for the *N*-carbamoylsarcosine amidase, which might be involved in the degradation of 1-methylhydantion to *N*-carbamoylsarcosine (7). Genes encoding glycine decarboxylase components (*gcvPHT*) and dihydrolipoamide dehydrogenase were also present in the genome of *T. creatinophila* strain KRE 4^T^. Creatine amidinohydrolase-encoding or creatine reductase-encoding genes were not detected.

### Accession number(s).

The whole-genome shotgun project has been deposited at DDBJ/ENA/GenBank under the accession number LTDM00000000. The version described here is the first version, LTDM01000000.
